# Transfusion of blood components in pediatric age groups: an evidence-based clinical practice guideline adapted for the use in Egypt using ‘Adapted ADAPTE’

**DOI:** 10.1007/s00277-024-05657-4

**Published:** 2024-02-22

**Authors:** Galila Mokhtar, Amira Adly, Ashraf Abdel Baky, Dina Ezzat, Gehan Abdel Hakeem, Hoda Hassab, Ilham Youssry, Iman Ragab, Ivan Florez, Laila M. Sherief, Magdy El-Ekiaby, Marwa Zakaria, Mervat Hesham, Naglaa Shaheen, Niveen Salama, Nouran Salah, Rasha A. A. Afifi, Rasha El-Ashry, Salwa Youssef, Seham Ragab, Sonia A. Habib, Tarek Omar, Yasser Amer, Yasser Wali, Sara Makkeyah, Egyptian Pediatric Clinical Practice Guidelines Committee (EPG)

**Affiliations:** 1 Pediatric Hematology and Oncology Unit, Department of Pediatrics, Ain Shams University, Cairo, Egypt; 2 Department of Pediatrics, Faculty of Medicine, Ain Shams University, Cairo, Egypt; 3 Department of Pediatrics, MTI University, Cairo, Egypt; 4 Department of Pediatrics, Armed Forces College of Medicine (AFCM), Cairo, Egypt; 5 Pediatric Hematology and Oncology Unit, Department of Pediatrics, Beni Suef University, Beni Suef, Egypt; 6 Pediatric Hematology and Oncology Unit, Department of Pediatrics, Minia University, Minia, Egypt; 7 Pediatric Hematology and Oncology Unit, Department of Pediatrics, Alexandria University, Alexandria, Egypt; 8 Pediatric Hematology and Oncology Unit, Department of Pediatrics, Cairo University, Giza, Egypt; 9 Department of Pediatrics, University of Antioquia, Medellin, Colombia; 10 Department of Pediatrics, McMaster University, Hamilton, ON Canada; 11 Pediatric Hematology and Oncology Unit, Department of Pediatrics, Zagazig University, Zagazig, Egypt; 12 Department of Clinical Pathology and Transfusion Medicine, Shabrawishi Hospital, Cairo, Egypt; 13 Pediatric Hematology Department, Misr Children’s Hospital, Health Insurance Organization, Cairo, Egypt; 14 Pediatric Hematology and Oncology Unit, Department of Pediatrics, Mansoura University, Monsoura, Egypt; 15 Department of Clinical Pathology and Transfusion Medicine, Ain Shams University, Cairo, Egypt; 16 Pediatric Hematology and Oncology Unit, Department of Pediatrics, Menoufia University, Menoufia, Egypt; 17 Pediatric Hematology and Oncology Unit, National Research Center, Giza, Egypt; 18 Department of Pediatrics, Faculty of Medicine, Alexandria University, Alexandria, Egypt; 19 Alexandria Center for Evidence-Based Clinical Practice Guidelines, Alexandria University, Alexandria, Egypt; 20 Department of Pediatrics, Quality Management Department, King Saud University Medical City, Riyadh, Saudi Arabia; 21 Pediatric Hematology/Oncology Unit, Child Health Department, Sultan Qaboos University Hospital, Muscat, Oman

**Keywords:** Transfusion, Pediatrics, Practice guideline adaptation

## Abstract

Pediatric transfusion is a complex area of medicine covering a wide age range, from neonates to young adults. Compared to adult practice, there is a relative lack of high-quality research to inform evidence-based guidelines. We aimed to adapt the pre-existing high-quality practice guidelines for the transfusion of blood components in different pediatric age groups to be available for national use by general practitioners, pediatricians, and other health care professionals. The guideline panel included 17 key leaders from different Egyptian institutions. The panel used the Adapted ADAPTE methodology. The panel prioritized the health questions and recommendations according to their importance for clinicians and patients. The procedure included searching for existing guidelines, quality appraisal, and adaptation of the recommendations to the target context of use. The guideline covered all important aspects of the indications, dosing, and administration of packed red cells, platelets, and fresh frozen plasma. It also included transfusion in special situations, e.g., chronic hemolytic anemia and aplastic anemia, management of massive blood loss, malignancies, surgery, recommendations for safe transfusion practices, and recommendations for modifications of cellular blood components. The final version of the adapted clinical practice guideline (CPG) has been made after a thorough review by an external review panel and was guided by their official recommendations and modifications. A set of implementation tools included algorithms, tables, and flow charts to aid decision-making in practice. This adapted guideline serves as a tool for safe transfusion practices in different pediatric age groups.

**Supplementary Information:**

The online version contains supplementary material available at 10.1007/s00277-024-05657-4.

## Adapted Source Guidelines

1. Guideline on the investigation and management of acute transfusion reactions Prepared by the BCSH Blood Transfusion Task Force. BSH 2012 [1]
2. Guidelines on red cell transfusion in sickle cell disease. BSH 2016a [2, 3]
3. Guidelines for the use of platelet transfusions. BSH 2016b [4].
4. Recommendations on Red Blood Cell Transfusion in General Critically Ill Children Based on Hemoglobin and/or Physiologic Thresholds from the Pediatric Critical Care Transfusion and Anemia Expertise Initiative. TAXI 2018 [5]
5. Clinical practice guidelines: Use of Blood Components in Newborns. NNF 2020 [6].
6. Guidelines on the use of irradiated blood components. BSH 2020a [7].
7. Guidelines on Transfusion for Fetuses, Neonates and Older Children. BSH 2016—addendum 2020b [8, 9]
8. British Society of Hematology Guidelines on the spectrum of fresh frozen plasma and cryoprecipitate products: their handling and use in various patient groups in the absence of major bleeding. BSH 2018 – addendum 2020 [10, 11].

## Introduction

Blood and blood products are currently incorporated into the World Health Organization (WHO) model list of essential medicines [12]. Blood transfusion represents an essential component of modern healthcare systems, and when used appropriately, it saves lives, improves health conditions, and enhances patients’ outcomes. However, improper or unnecessary use can increase the risk of serious, acute, and delayed adverse effects, which constitutes a burden, especially in underprivileged settings (Implementation tools Figure S1).

Pediatric transfusion is a complex area of medicine covering a wide age range, from intrauterine life to young adults. The prescriber must balance the risks and benefits of transfusion in each age group and be aware of the indications for special components. Compared to adult practice, there is a relative lack of high-quality research to inform evidence-based guidelines [13]. Evidence-based literature on pediatric transfusion practices is limited, particularly for non-red blood cell products, and many recommendations are extrapolated from studies in adult populations [14]. Recognition of these knowledge gaps has led to an increasing number of clinical trials focusing on children and the establishment of pediatric transfusion working groups in recent years.

Clinical Practice Guidelines (CPGs) are statements that include recommendations intended to optimize patient care, which are informed by a systematic review of evidence and an assessment of the benefits and harms of alternative care options [15]. CPGs have been identified as one of the main tools for improving evidence-based healthcare quality and safety. In healthcare settings with limited resources, adaptation of CPGs is a viable and effective alternative to de novo development of CPGs. [16, 17]

The Egyptian Pediatric CPGs Committee (EPG) is the Country’s first national initiative for the production of pediatric evidence-based CPGs that started in 2018 using one of the CPG formal adaptation methodological frameworks: The Adapted ADAPTE. [18] The aim of this adapted clinical practice guideline (CPG) is to provide evidence-based recommendations for the transfusion of blood components in the pediatric age group.

## Methods

The Adapted ADAPTE CPG formal adaptation method included three phases (setup, adaptation, and finalization), nine modules, and 24 steps with modifications in the steps and tools to suit the local general healthcare setting in Egypt [16]. It was registered on the PREPARE (Practice Guideline Registration for Transparency) with the registration number PREPARE-2022CN444 [19]. The Egyptian Pediatric Clinical Practice Guidelines Committee (EPG) Hematology Group [20] included 17 members with recognized clinical and research expertise in pediatric hematology, representing ten research centers from different Egyptian Institutions (Ain Shams University, Alexandria University, the Armed Forces College of Medicine, Beni-Suef University, Cairo University, the Health Insurance Organization, Mansoura University, Minia University, the National Research Center, and Zagazig University). Three experts in guideline methodology supervised the guideline adaptation process. The panel chose the high-priority health topic “transfusion of blood components in the pediatric age groups” due to its clinical importance. Identification and formulation of the health questions were done using the PIPOH (Population / Intervention / Professionals / Outcomes / Healthcare settings) model.

We performed a literature search of the relevant CPG and bibliographic electronic databases PubMed in March 2022 for the relevant source original CPGs retrieval. The following search terms were used: “transfusion” “neonates” “pediatrics”, “red cell” “fresh frozen plasma”, “platelets”, “blood component modification” and “acute transfusion reactions”. Corresponding MeSH terms were used, in addition to the search for titles and abstracts.

Inclusion and exclusion criteria were identified. The search was restricted to CPGs published from January 2012 to January 2022 to capture consensus guidelines in the last 10 years. The following filters were applied: humans, English, and consensus guidelines. Other types of research articles, like reviews of literature, were not included.

The search and screening results revealed eight source CPGs that were assessed for methodological quality by six independent raters using the Appraisal of Guidelines for Research and Evaluation (AGREE) II Instrument [21, 22]. Eight guidelines received an AGREE II Domain 3 (Rigor of development) and an Overall Assessment 1 score of more than 70%; hence, they were included as sources for the adaptation. Official adaptation request approval was obtained from the selected guidelines authors. This was followed by phrasing of the health questions that needed to be tackled and then drafting the adapted CPG. The external review process included consultation with three national and one international external reviewer and the finalization of the adapted CPG with a set of implementation tools. The EPG Hematology Group will review the need for updates every 5 years.

Furthermore, our CPG group opted to report the components of the adapted CPG using the extension of the Reporting Items for Practice Guidelines in Healthcare (RIGHT) Statement for the reporting of adapted CPGs (RIGHT-Ad@pt Tool) [23]. (Supplementary file).

## Results

### Recommendations

#### Part I: Transfusion in neonates

Neonates, especially extremely low birth-weight infants, are among the groups of patients undergoing transfusions frequently [24]. Transfusion thresholds vary according to the clinical context, including the gestational age at birth. Neonates are exposed to higher specific transfusion risks compared to other age groups; hence, many special aspects must be considered for transfusion therapy in neonates.

#### Pre-transfusion testing

##### NT.1. What are the samples required for pre-transfusion testing in neonates?

Within the first 4 months, wherever possible, samples from both mother and infant should be obtained for initial ABO and D group determination. The antibody screen should be undertaken on the maternal sample when available. A maternal sample is preferred for antibody testing for the following reasons:

- If maternal antibody has bound to fetal cells in vivo, the resulting lower concentration of antibody in neonatal plasma could lead to a false negative antibody screen result.
- It is easier to obtain a sufficiently large sample from the mother to allow for screening and antibody identification if required.
- Sample collection from the infant exacerbates the anemia of prematurity.

The maternal sample should be collected within 3 days predelivery or collected post-delivery.

(GPP)

#### Red cell transfusion

##### NR.1. What are the indications and thresholds for transfusion of packed red cells in acutely ill neonate without other comorbid conditions?

- Studies to date support restrictive transfusion thresholds (2B) and suggested Hb thresholds for top-up transfusions are given in Table 1.

**Table 1:** Suggested transfusion thresholds for preterm neonates*

	Suggested transfusion threshold Hb (g/dl)		
Postnatal age	Ventilated	On oxygen/NIPPV	Off oxygen
First 24 h	< 12	< 12	< 10
< week 1 (d 1–7)	< 12	< 10	< 10
Week 2 (d 8–14)	< 10	< 9.5	< 7.5†
> week 3 (d15 onwards)	< 10	< 8.5	< 7.5†

*NIPPV* non-invasive positive pressure ventilation

*Standard definition of preterm is <37 weeks gestational age at birth but table applies to very preterm neonates (<32 weeks)

†It is accepted that clinicians may use up to 85 g/dl depending on the clinical situation

Table 1 does not include suggested thresholds for moderate to late preterm (≥32 weeks gestational age at birth) or term neonates, as there is little evidence regarding the appropriate thresholds for these groups. Clinicians may consider similar thresholds to those used for preterm babies off oxygen.

##### NR.2. What are the indications for transfusing packed red cells in acute ill neonate with neonatal sepsis?

- The decision to transfuse is based on the clinical status. For those not requiring cardiopulmonary support or oxygen supply and whose condition is stable, transfusion is not usually required unless Hb level is below 7g/dl. (GPP)

##### NR.3. What is the volume required for red cell transfusion in neonates?

- Transfusion volumes of 15 ml/kg are generally recommended for non-bleeding neonates. (2C)
- Repeated small-volume ‘red cell transfusions (up to 20 ml/kg) are commonly carried out in preterm babies, mainly to replace losses from repeated blood testing exacerbated by reduced red cell production (anemia of prematurity). (2B)

##### NR.4. What are the characteristics of red cells used for exchange transfusion in neonates?

- A specific red cell component for neonatal exchange transfusion, usually group O, and should also be compatible with any maternal antibody. (GPP)
- Red cells suitable for neonatal exchange need to be irradiated and ‘fresh’ (before the end of Day 5 following donation, with a 24-h shelf-life post-irradiation) to reduce the risk of recipient hyperkalemia. They have a controlled hematocrit 0.5–0.6. They are negative for high-titre anti-A and anti-B antibodies. (GPP)
- Exchange blood transfusion (EBT) should not be undertaken with red cells straight from 4 °C storage, and an approved blood-warming device can be used to avoid hypothermia. The use of a blood warmer is only appropriate if the infusion is given at a constant rate (warming is not suited to the intermittent bolus nature of a single-vessel EBT where the ‘push–pull’ cycle method is used). Blood warming during EBT should not be uncontrolled, e.g., infusion lines exposed to a radiant heater, because of the risk of red cell hemolysis. (GPP)

#### Platelet transfusion

##### NP.1. What are the indications and thresholds for platelet transfusion in neonates?

*In preterm neonate*

- For preterm neonates with very severe thrombocytopenia (Platelet count below 25 × 10.^9^/l) platelets should be administered in addition to treating the underlying cause of the thrombocytopenia. (2C)

*In full-term neonate*

- For non-bleeding neonates, platelet transfusions should not be routinely administered if platelet count is ≥ 25 × 10.^9^/l. (1B)
- If Platelet count < 25 × 10.^9^/l transfuse in Neonates with no bleeding. (2C)
- If platelet count < 50 × 10.^9^/l transfuse in Neonates with bleeding, current coagulopathy, before surgery, or infants with fetal-neonatal alloimmune thrombocytopenia (FNAIT) if previously affected sibling with intracranial hemorrhage (ICH). (2C)
- If platelet count < 100 × 10.^9^/l transfuse in Neonates with major bleeding or requiring major surgery (e.g., neurosurgery). (2C)

*In antibody-mediated thrombocytopenia*

- In fetal-neonatal alloimmune thrombocytopenia (FNAIT), maintaining platelet count > 30 × 10.^9^/l is strongly recommended. (1D)

*In preterm neonate with patent ductus arteriosus (PDA)*

- The routine use of platelet transfusion for PDA closure in thrombocytopenic preterm neonates with PDA is not recommended. (2C)
  (See implementation tools Figure S2).

##### NP.2. What is the dose and rate for platelet transfusion in neonates?

- Typical transfusion volume: 10–20 ml/kg. Transfusion rate: 10–20 ml/kg/h. (GPP)

##### NP.3. Is ABO compatibility required for platelet transfusion in neonates?

- ABO, Rh-matched platelets should be used when available to maximize increments. (2C)
- It is acceptable to use ABO incompatible platelets to reduce wastage. Platelets tested and negative for high titre hemagglutinins and non-group O platelets are associated with a lower risk of hemolysis. (1B)

#### Fresh frozen plasma

##### NF.1. What are the therapeutic indications of fresh frozen plasma (FFP) transfusion in neonates?

- FFP may be of benefit in neonates with clinically significant bleeding (including massive blood loss) or prior to invasive procedures with a risk of significant bleeding, and who have an abnormal coagulation profile, defined as a PT or aPTT significantly above normal gestational and postnatal age-related reference range. (Considering local reference ranges where available). (2C)
- FFP is appropriate for the early management of severe hereditary protein C deficiency but should not be used in preference to protein C concentrate if this is available. (2B)
- FFP should be used for the management of severe hereditary protein S deficiency. (2B)
- Management of DIC, inherited deficiency of clotting factors, vitamin- K deficiency bleeding (prothrombin complex concentrates are preferable to FFP). (2C)
- If virally inactivated specific clotting factors are not available, pathogen-reduced plasma may be used for factor replacement in congenital coagulation factor deficiency. (1C)
- FFP transfusion is preferred over cryoprecipitate in the management of disseminated intravascular coagulation. (2C)
- Where indicated, cryoprecipitate may be used if there is persistent hypofibrinogenemia (< 1.0 g/L) despite FFP transfusion, or in conjunction with FFP for very low or rapidly falling fibrinogen. (2C)

##### NF.2. What are the prophylactic indications of fresh frozen plasma transfusion in neonates?

- The routine use of prophylactic FFP in preterm neonates is not recommended. (1C)
- Prophylactic FFP is not recommended in non-bleeding neonates receiving therapeutic hypothermia and having deranged coagulation parameters. (1B)
- Neonates with deranged coagulation parameters and planned for surgical or invasive procedures should receive FFP. (1D)

##### NF.3. What are the indications of fresh frozen plasma transfusion in neonatal emergency?

- FFP 15–20 ml/kg given 8–12 hourly may be used as first line therapy to treat acquired neonatal purpura fulminans in association with protein C or protein S deficiency while the underlying cause is being investigated. The underlying cause should be treated, and it may be helpful to monitor protein C / Protein S (PC/PS) levels. (GPP)

##### NF.4. What are the contraindications to plasma transfusion in neonates?

- There is no evidence to support the routine use of FFP to try to correct abnormalities of the coagulation screen alone in non-bleeding neonates. (2C)
- Prophylactic FFP is not recommended in non-bleeding neonates receiving therapeutic hypothermia and having deranged coagulation parameters. (1B)
- FFP should not be used in the management of inherited factor deficiencies other than in a few exceptional circumstances where specific factor concentrates are not available. (1B)
- FFP should not be used for simple volume replacement or routinely for prevention of intraventricular hemorrhage. (2C)
- FFP should not be used for performing a partial exchange transfusion for polycythemia. (GPP)

##### NF.5. What is the dose and rate of plasma transfusion in neonates?

- For patients who have abnormal clotting tests and other factors (i.e., personal/family bleeding history, drug history, bleeding risk associated with planned procedure or thrombocytopenia) that indicate a significant bleeding risk during a procedure, then a starting dose of 15 ml/ kg of FFP can be considered. (1B)
- For the first 15 min: 1 mL/kg/h.; reassess patient, if well tolerated increase rate as per Physician’s order (this is recommended “test dose, slow rate of infusion”). Usual rate: 10 to 20 mL/kg/hr. (GPP)

## Part II: Transfusion in infants, children, and adolescents

Blood transfusion plays an important role in the treatment of sick children. A blood product transfusion may be required to treat acute blood loss, or hypoproliferative bone marrow states, as in bone marrow failure, cancer or other causes of bone marrow suppression. To transfuse a blood component, the expected benefits to the recipient should outweigh the potential risks.

### Packed red blood cells (PRBCs)

#### I. Indications

##### PR.1. What are the indications and thresholds of red cell transfusion in different pediatric diseases?

In children with cancer

- There is insufficient evidence to make recommendations for pre-transfusion Hb thresholds in pediatric hematology/ oncology patients and those undergoing stem cell transplantation. (2C)
- In children with oncologic diagnoses who are critically ill or at risk for critical illness, and hemodynamically stable, a Hb concentration of 7–8 g/dl is suggested as a threshold for RBC transfusion. (2C)

In children with pure red cell aplasia

- Patients with chronic anemia due to red cell aplasia may require a Hb threshold of 8 g/dl. (2C)

In children with sickle cell disease

- Transfusion is recommended and may be lifesaving in acute sickle complications such as splenic sequestration, hepatic sequestration, aplastic crisis and severe acute chest syndrome. (1B)
- Simple transfusion to steady state hemoglobin concentration is indicated for patients with acute exacerbation of anemia as a result of aplastic crisis or sequestration crisis. (1B)
- Over-transfusion (to Hb > 8 g/dl) should be avoided in sequestration crises because of the risk of hyperviscosity due to the re-entry of sequestered red cells into the circulation. (1C)
- There is no evidence that transfusion shortens the duration of a painful crisis. Transfusion is not recommended in uncomplicated painful crises but should be considered if there is a substantial drop in Hb from baseline (e.g., > 2 g/dl or to Hb < 5 g/dl), hemodynamic compromise, or concern about impending critical organ complications. (1C)
- Transfusion should be considered in the unwell patient with acute multi-organ failure, mesenteric syndrome (1C), and patients with severe sepsis (2C).
- Transfusion is recommended in cases of acute chest syndrome with hypoxia. Transfusion may be given by simple or exchange transfusion depending on clinical severity under the guidance of the specialist hemoglobinopathy team. (1B)
- Adults or children with signs or symptoms suggestive of acute ischemic stroke should be transfused to sickle hemoglobin (HbS) < 30% pending further investigation. Those with confirmed stroke due to sickle cell disease should continue regular transfusions indefinitely. (1B)
- Transfusion is not recommended to treat steady state anemia provided that Hb has not fallen over a period of time to symptomatic levels (e.g., with developing chronic kidney disease). (1C)

In critically ill children admitted to PICU

- In critically ill children or those at risk for critical illness, who are hemodynamically stable and who have an Hb concentration ≥ 7 g/dl, we recommend not administering a RBC transfusion. (1B)
- When deciding to transfuse an individual critically ill child, consider not only the hemoglobin (Hb) concentration, but also the overall clinical context (e.g., symptoms, signs, physiological markers, laboratory results) and the risk, benefits, and alternatives to transfusion. (GPP)
- In critically ill children or those at risk for critical illness, we recommend measuring the hemoglobin (Hb) concentration before prescribing each RBC transfusion; knowledge of Hb concentration is not required before RBC transfusion if the patient has life threatening bleeding. (GPP)

In the preoperative setting

- A perioperative Hb transfusion threshold of 7 g/dl should be used in stable patients without major co-morbidity or bleeding. (1C)

##### PR.2. What are the definitions and precautions of transfusion with massive blood loss?

- Massive blood loss (MBL) may be defined as either 80 ml/kg in 24 h, 40 ml/kg in 3 h, or 2–3 ml/kg/min. In clinical practice, hemodynamic changes compatible with hypovolemia accompanying evidence or suspicion of serious hemorrhage are the usual triggers. (GPP)

Key principles in MBL are:

1. Early recognition of children at risk of MBL using clinical parameters
2. Education of staff to understand when to activate/trigger the local major hemorrhage protocol.
3. Active resuscitation and control of bleeding
4. Seek specialist assistance.
5. Rapid provision of O RhD-negative or group specific red cells
6. Prescribe all transfused components in ml/kg bodyweight (for children < 50 kg) and not as units.
7. Anticipate and treat coagulopathy and thrombocytopenia in trauma with early use of FFP and consideration of platelets and cryoprecipitate in on-going bleeding.
8. Use tranexamic acid in trauma.
9. Avoid hypothermia, hypocalcemia, acidosis, and hyperkalemia.

Appropriate aliquots to be transfused are as follow:

- RBCs 20 ml/kg aliquots (maximum four adult units), RhD-negative or ABO and RhD-specific (ideally, cross-matched).
- Group specific FFP in 20 ml/kg aliquots (maximum four adult units).
- Platelets in 15–20 ml/kg aliquots (maximum one adult therapeutic dose) to be considered after every 40 ml/kg RBCs transfusion.
- Cryoprecipitate 10 ml/kg (maximum two pools).

Initial immediate transfusion of 20 ml/kg RBCs should be given (up to four adult units).

There was no difference between early administration of plasma, platelets, and RBCs in a 1:1:1 ratio and in a 1:1:2 ratio^25^. A ratio of at least 1 FFP:2 RBC is recommended in early resuscitation of major hemorrhage (in major trauma clinicians may consider aiming for a ratio of 1 FFP:1 RBC). Platelets and cryoprecipitate must be considered if active bleeding persists after initial resuscitation.

These aliquots should be repeated in recommended ratios as necessary until bleeding is controlled. Ratios should be modified accordingly once laboratory parameters are available.

The therapeutic aims should be Hb 8 g/dl, fibrinogen > 1.5 g/l, PT ratio < 1.5, platelet count > 75 × 10^9^/l. Careful monitoring for the adequacy of resuscitation and for circulatory overload is essential.

#### II. Volume and rate of PRBCs transfusion

##### PR.3. What is the best way of ordering volume of PRBCs in children?

- Prescription of blood components for pediatric transfusion should be in milliliters unless there are local risk-assessed protocols for prescribing in units for older children, and the maximum volume should not be greater than prescribed for adults. (1C)

##### PR.4. What is the volume and rate of PRBCs transfusion in acute ill children?

In a non-bleeding infant or child, it is important to take into account the pre-transfusion Hb in relation to the transfusion threshold, and it is recommended that a post-transfusion Hb no more than 2 g/dl above the threshold be aimed for.

$$Volume\;to\;tranfuse\left(ml\right)=Desired\;Hb\left(\frac g{dl}\right)-actual\;Hb\left(\frac g{dl}\right)\;\times\;weight(kg)\times40/10$$

Transfusion rate 5 ml/kg/h (usual maximum rate: 150 ml/h) (GPP)

##### PR.5. What is the transfusion volume for regular transfusion in transfusion-dependent thalassemia in chronic transfusion program?

 (Desired − actual Hb (g/dl)) × weight (kg) × 3 = mls to be transfused assuming the hematocrit of the unit is 0.58. (Table 2) (GPP)

**Table 2:** Suggested volume of blood to transfuse in transfusion dependent thalassemia

		Hematocrit of donor red cells			
Target increase in hemoglobin level		50%	60%	75%	80%
	2 g/dl	12 ml/kg	10 ml/kg	8 ml/kg	7.5 ml/kg
	3 g/dl	18 ml/kg	15 ml/kg	12 ml/kg	11.2 ml/kg
	4 g/dl	24 ml/kg	20 ml/kg	16 ml/kg	15 ml/kg

##### PR.6. What are the precautions for PRBCs transfusion in transfusion-dependent thalassemia?

- Blood must be ABO compatible, and antigen negative for any clinically significant antibodies the patient is known to have, or to have had previously identified, even if they are not currently detectable. It should be fully matched for all the Rh antigens and K.
- Units should be less than 2 weeks old and, in adults, of larger volume where possible.
- There should be a clear record of the patient’s transfusion requirements, outlining volume, frequency, and target hemoglobin.
- Transfusions will be given on each occasion in a designated age-appropriate area with suitable facilities, experienced, regular named nurses, and familiar supervising medical team.
- Pre-arranged transfusions should be started within 30 min of the patient’s arrival.
- (GPP)Patients with transfusion-dependent anemia and sickle cell disease should preferably have extended red cell phenotyping or genotyping (D, C, c, E, e, K, Fya, Fyb, Jka, Jkb, M, N, S, and s) prior to transfusion and, as a minimum, red cells should be matched for Rh (D, C, c, E, e) and K antigens. (GPP)
- Pre-storage leukodepletion of cellular blood products achieving a residual leukocyte count < 5 × 10. [6] per unit allows the reduction of CMV transmission to a level at least equivalent to the transfusion of sero-negative blood components for those patients at major risk of severe CMV transfusion-associated disease. (GPP)

#### Platelets

Platelet concentrates can be obtained from whole blood collections either via the buffy coat (BC) or platelet-rich plasma (PRP) manufacturing processes. Apheresis platelets (A-PC), on the other hand, are collected from a single donor by using an apheresis machine with an integrated leukoreduction system (removing ≥ 99% of white cells). [26, 27]

Although several studies have shown a higher in vivo corrected count increments (CCIs) with the transfusion of apheresis platelets compared to BC- or PRP-PCs, however, a higher CCI may not result in an improved hemostatic effect or bleeding prevention [28]. The risk of bacterial contamination is theoretically lowest in apheresis PC, since the procedure implies only one donor and one venipuncture, in contrast to pooled PC, where four to six donors and venipunctures are necessary. However, the estimated contamination rates were highly variable in different studies, depending on the time of sampling and the bacterial detection method. [27, 29–32]

The shortage of platelets in many centers makes the use ABO-incompatible platelet an accessible and acceptable choice. There is no defined critical Anti-A/B titre that will predict in vivo hemolysis, and the cutoff values in use range from 32 to 200 for IgM anti-A (tube saline) technique and 256 to 512 for IgG anti-A (IAT). [33]

##### PP.1. What are the indications for platelet transfusion in infants and children during acute bleeding?

*In non-immune thrombocytopenia*

- In severe bleeding, maintain the platelet count above 50 × 10.^9^ /l. Consider empirical use for the initial management of major hemorrhage. (1C)
- In patients with multiple trauma, traumatic brain injury or spontaneous intracerebral hemorrhage, maintain the platelet count above 100 × 10.^9^ /l. (2C)
- In patients with bleeding that is not considered severe or life-threatening, consider platelet transfusion if the platelet count is below 30 × 10.^9^ /l. (2C)

*In immune thrombocytopenia*

- In ITP, consider co-administration of intravenous immunoglobulin in addition to the platelet transfusion. (2C)
- In post-transfusion purpura (PTP), intravenous immunoglobulin is the treatment of choice. (1C)
- Give therapeutic platelet transfusions (more than one dose) to treat serious bleeding. (1C)
- Only use platelet transfusion prior to a procedure or surgery when other treatment has failed and/or the intervention is urgent. Usual threshold counts may be unachievable or unnecessary and individual case review is required. (1C)

*In platelet function disorders (congenital)*

- If pharmaceutical therapies are contraindicated, ineffective or if there is high risk of bleeding, consider transfusion of platelets. In Glanzmann thrombasthenia, consider human leucocyte antigen (HLA)-matched platelets (2C) where available (GPP).

*In drug-induced platelet function disorders (acquired)*

- Do not use platelet transfusion pre-procedure when antiplatelet agents have not been discontinued. (2C)
- Use general hemostatic measures to treat bleeding in patients during treatment with aspirin, P2Y12 antagonists, or glycoprotein IIa/IIIb inhibitors. If necessary, consider drug cessation and reversal of the effect of co-prescribed anticoagulants. (2C)
- Use TXA to counteract the effect of anti-platelet agents when a risk/benefit assessment would support this. (1B)
- Consider the use of platelet transfusion as an additional measure to those suggested above for critical bleeding. (2C)

##### PP.2. What are the indications for prophylactic platelet transfusion?

*In critically ill child*

- Use the platelet count thresholds for reversible bone marrow failure as a general guide for prophylactic platelet transfusion in patients with critical illness in the absence of bleeding or planned procedures. (2C)

*In autoimmune thrombocytopenia (ITP)*

- Do not use prophylactic platelet transfusions in patients with autoimmune thrombocytopenia. (1C)

*In reversible bone marrow failure (receiving intensive chemotherapy or undergoing allogeneic hematopoietic stem cell transplant (HSCT)*

- Give prophylactic platelet transfusions to patients to maintain a platelet count at or above 10 × 10.^9^/l. (1B)
- Use only one adult dose (one unit) routinely for prophylactic platelet transfusions. (1A)
- Consider increasing the threshold for prophylactic platelet transfusion to between 10 and 20 × 10.^9^/l /l in patients judged to have additional risk factors for bleeding. Individual review is required. (2C)

*In chronic bone marrow failure (when recovery is not anticipated)*

- Use a ‘no prophylactic platelet transfusion’ strategy for asymptomatic patients with chronic bone marrow failure. (2B)
- Give prophylactic platelet transfusions to patients with chronic bone marrow failure receiving intensive treatment. (1B)
- Manage patients with chronic bleeding of WHO grade 2 or above individually *(see implementation tools)*, according to the severity of their symptoms and signs. Consider a strategy of prophylaxis (e.g., twice a week). (2C)

*Prior to procedures or surgery:*

- Do not give platelet transfusions routinely prior to:
- bone marrow aspirate or trephine biopsy. (1B)
- peripherally inserted central catheters (PICCs). (2C)
- traction removal of tunneled CVCs. (2C)
- cataract surgery. (2C)
- Whenever possible use a procedure/equipment associated with the lowest bleeding risk. Apply local measures, such as compression, to reduce the risk of bleeding post-procedure. (1C)

*Prior to procedures or surgery*

Consider performing the following procedures above the platelet count threshold indicated:

- venous central lines (both tunneled and un-tunneled), inserted by experienced staff using ultrasound guidance techniques, when the platelet count is > 20 × 10.^9^ /l. (1B)
- lumbar puncture when the platelet count is ≥ 40 × 10.^9^/l. (2C)
- insertion/removal of epidural catheter when the platelet count is ≥ 80 × 10.^9^/l. (2C)
- major surgery—when the platelet count is > 50 × 10.^9^/l. (1C)
- neurosurgery or ophthalmic surgery involving the posterior segment of the eye when the platelet count is > 100 × 10.^9^ /l. (1C)
- percutaneous liver biopsy when the platelet count is > 50 × 10.^9^/l. Consider trans-jugular biopsy if the platelet count is below this level. (2B)

##### PP.3. What is the dose and rate for platelet transfusion?

Typical transfusion volume: 10–20 ml/kg for children < 15 kg, or a single pack for children ≥ 15 kg, maximum volume 1 pack. Transfusion rate: 10–20 ml/kg/h. (GPP).

NB: one unit of platelets is around 3 × 10^11^ platelets.

##### PP.4. What are the contraindications of platelet transfusion?

- In patients with thrombotic microangiopathies only use platelet transfusions to treat life-threatening bleeding. (1C)

##### PP.5. How to avoid/ manage the risks of platelets transfusion?

- Hospitals should establish a strategy to maximize the transfusion of ABO compatible platelets, especially to patients who require regular platelet support. (2B)
- ABO, Rh matched platelets should be used when available to maximize increments. (2C)
- It is acceptable to use ABO incompatible platelets to reduce wastage. Platelets tested and negative for high titre hemagglutinins and non-group O platelets are associated with a lower risk of hemolysis. Pooled platelets suspended in Platelet Additive Solution (PAS) would also be expected to reduce this risk. (1B)
- RhD negative girls or women of childbearing potential should receive RhD negative platelets. If unavailable, RhD positive platelets can be given with anti-D prophylaxis. (1B)
- For RhD negative boys under 18 years of age, those who already have anti-D antibodies, and transfusion-dependent adults, the platelets of choice are RhD negative. RhD positive platelets should be given if RhD negative platelets are unavailable or to prevent wastage of RhD positive components. Anti-D prophylaxis is not required. (1B)
- In patients with a history of allergic transfusion reactions, apart from mild, use platelets suspended in PAS. If reactions continue or are severe, washed platelets (resuspended in 100% PAS) may be required. (1B)
- Patients with hypoproliferative thrombocytopenia who are refractory to platelet transfusions and have class I HLA antibodies should receive class I HLA-selected platelet transfusion. (2C)
- Patients with hypoproliferative thrombocytopenia who continue to be refractory to HLA-selected platelet transfusions and have human platelet antigen (HPA) antibodies should receive HPA-selected platelet transfusion. (2C)
- Patients with hypoproliferative thrombocytopenia who are not refractory to platelet transfusion should not receive HLA-selected or HPA-selected platelets. (2C)
- (See implementation tools Figure S3 for approach to platelet refractoriness)

#### Fresh frozen plasma

##### PF.1. What are the indications for plasma transfusion during acute bleeding in pediatrics?

- Pathogen-reduced plasma may be used for factor replacement in congenital coagulation factor deficiency if virally inactivated specific clotting factors are not available. (1C)
- FFP may be beneficial in children with DIC who have a significant coagulopathy (PT/APTT > 1.5 times midpoint of normal range or fibrinogen < 1.0 g/l) associated with clinically significant bleeding or prior to invasive procedures. (2C)
- In DIC, cryoprecipitate may be given if the fibrinogen is < 1.0 g/l despite FFP, or in conjunction with FFP for very low or rapidly falling fibrinogen. (2C)
- Prophylactic FFP should not be administered to non-bleeding children with minor prolongation of the prothrombin time (2B)/ activated partial thromboplastin time including prior to surgery, although it may be considered for surgery to critical sites (2C)
- Prophylactic cryoprecipitate should not be routinely administered to non-bleeding children with decreased fibrinogen including prior to surgery. It may be considered for fibrinogen < 1 g/l for surgery at risk of significant bleeding or to critical sites. (1C)
- FFP should not be used in the management of inherited factor deficiencies other than in a few exceptional circumstances where specific factor concentrates are not available. (1C)
- Urgent plasma exchange with solvent detergent fresh frozen plasma (SD FFP) is indicated for thrombotic thrombocytopenic purpura (TTP) (1B) and some forms of atypical hemolytic uremic syndrome (HUS) (2C)

##### PF.2. What is the dose/ frequency for plasma transfusion during acute bleeding in pediatrics?

- The recommended therapeutic dose of FFP is 10–15 ml/kg of body weight given 8–12 hourly depending on the clinical situation and laboratory parameters. (1C)

##### PF.3. What is the best choice of blood group for plasma transfusion?

- Plasma of donors with identical ABO blood group to the recipient should be used as the first choice. If this is not possible, ABO non-identical but compatible plasma is acceptable if it has ‘low-titre’ anti-A or anti-B activity. (1B)
- Group O plasma should only be given to group O patients. (1B)
- Fresh frozen plasma and cryoprecipitate of any RhD group may be transfused. If RhD positive plasma is given to an RhD negative individual, no anti-D prophylaxis is required. (1B)

#### Part III: Modification of blood components and related precautions

Blood components are foreign to the recipient and may produce adverse effects, ranging from mild allergic manifestations to fatal reactions. To avoid and reduce such complications, blood products can be modified. Common modifications methods include leukoreduction, irradiation, and washing.

Leukoreduction of blood components can be done either pre-storage at the time of collection and processing, post-processing (within the blood bank), or by the side of the patient (post-storage) [34]. Pre-storage leukoreduction is the standard of care in many areas of the world, with the advantages of eliminating inflammatory cytokine responses for transfusion reactions [35]and minimizing HLA-alloimmunization [36] and some viral transmission [37]. It can be done by leukofilters (third generation), and components collected through apheresis devices meet the current standards of leukocyte depletion, that is, < 5 × 10^6^ WBC/unit of the blood component.^34^

##### MO.1. What are the indications and dose for irradiation of cellular blood components?

- Gamma- or X-irradiation of blood components, by validated systems, is the recommended procedure to prevent TA-GvHD. (1B)
- The minimum dose achieved in the irradiation volume should be 25 Gy, with no part receiving > 50 Gy. (1B)

In intrauterine transfusion and neonatal transfusion

- Red cells for neonatal exchange blood transfusion (EBT) should be irradiated. (1C)
- Routine irradiation of red cells for transfusion to preterm or term infants (other than for EBT) is not required unless there has been a previous intrauterine transfusion (IUT), in which case irradiated components should be administered until 6 months after the expected delivery date (40 weeks gestation). (2C)
- Routine irradiation of platelet transfusions for preterm or term infants is not required unless there has been a previous IUT, in which case irradiated components should be administered until 6 months after the expected delivery date (40 weeks gestation). (2C)

In first and second-degree relative

–All transfusions of cellular components and fresh plasma from first or second-degree relatives should be irradiated, even if the patient is immunocompetent. All HLA-selected components should be irradiated even if the patient is immunocompetent. (1B)

In immunodeficiency

- All severe congenital T-lymphocyte immunodeficiency syndromes with significant qualitative or quantitative T-lymphocyte deficiency should be considered indications for irradiation of cellular blood components. (1B)
- Once a diagnosis of severe T-lymphocyte immunodeficiency has been suspected, irradiated components should be given while further diagnostic tests are being undertaken. (1C0
- There is no indication for irradiation of cellular blood components for infants or children with temporary defects of T-lymphocyte function as the result of a viral infection. There is also no indication for irradiation of cellular blood components for adults or children who are HIV-antibody positive or who have acquired immune deficiency syndrome (AIDS). (1B)

In hematopoietic stem cell transplant (HSCT)

- All recipients of allogeneic HSCT should receive irradiated blood components from the time of initiation of conditioning chemo/ radiotherapy. The recommendation applies for all conditions where HSCT is indicated regardless of the underlying diagnosis. (1B)

Irradiated components should be continued until all of the following criteria are met:

1.  > 6 months have elapsed since the transplant date.
2. The lymphocyte count is > 1 × 10^9^/l.
3. The patient is free of active chronic GvHD.
4. The patient is off all immunosuppression.
5. If chronic GvHD is present or continued immunosuppressive treatment is required, irradiated blood components should be given indefinitely. (2C)
6. Allogeneic cellular blood components transfused to bone marrow and peripheral blood stem cell donors of all ages within 7 days prior to or during the harvest should also be irradiated. (2C)
7. Patients undergoing bone marrow or peripheral blood stem cell collections for future autologous re-infusion should receive irradiated cellular blood components for 7 days prior to and during the bone marrow/stem cell harvest to prevent the collection of viable allogeneic T lymphocytes, which can potentially withstand cryopreservation. (1C)
8. All patients undergoing autologous stem cell transplant (ASCT) irrespective of underlying diagnosis or indication for this treatment should receive irradiated cellular blood components from initiation of conditioning chemo/radiotherapy until 3 months post-transplant (6 months if total body irradiation was used in conditioning) unless conditioning, disease, or previous treatment determines indefinite duration, for example previous diagnosis of Hodgkin lymphoma (HL) or previous purine analogue treatment. (1C)

In aplastic anemia

- For patients with aplastic anemia, transfusion of irradiated cellular components is not routinely recommended, except for HLA-selected platelets, transfusion of granulocytes, donations from first- or second- degree relatives, or planned relevant treatment (e.g., ATG, alemtuzumab, HSCT). (1B)

In Hodgkin lymphoma (HL)

- All adults and children with HL at any stage of the disease should have irradiated red cells and platelets indefinitely. (2C)

Medications

- All patients treated with purine analogue drugs (fludarabine, cladribine, bendamustine, and pentostatin) should receive irradiated blood components indefinitely. (2C)
- Patients with hematological diagnosis treated with alemtuzumab should receive irradiated components. (2C)
- Patients with aplastic anemia undergoing treatment with ATG or alemtuzumab should receive irradiated blood components. (2C)
- Patients receiving ATG or other T-lymphocyte-depleting serotherapy for rare types of immune dysfunction conditions should receive irradiated blood components. (2C)
- Treatment of patients with rituximab is not an indication for use of irradiated cellular blood components unless this is indicated for a different reason (underlying diagnosis, type of component, or previous treatment). (1B)

In solid organ transplantation (SOT)

- Use of irradiated cellular blood components is not indicated for patients undergoing solid organ transplantation (SOT) who have received alemtuzumab or ATG as induction therapy or for treatment of graft rejection. (1B)

##### MO.2. What are the indications for use of washed PRBCs?

- For patients with recurrent febrile reactions, it is recommended to use a trial of premedication with oral paracetamol given 1 h before the reaction is anticipated (or non-steroidal anti-inflammatory drugs in patients with predominant chills or rigors—but an assessment of the risks of medication against the severity of reaction should be made in each case). Patients who continue to react should have a trial of washed blood components. (2C)
- Patients with recurrent or severe allergic or febrile reactions to red cells, and severely IgA-deficient patients with anti-IgA antibodies for whom red cells from an IgA-deficient donor are not available. (GPP)

##### MO.3. What are the indications to give leuko-filtered blood components in neonates?

- Provision of CMV safe blood for transfusion in preterm neonates by using CMV seronegative donors or leukoreduction or a combination of both is strongly recommended. (1C)
- For intrauterine transfusions use of CMV negative and leuco-depleted packed red blood cell is strongly recommended. (1C)

##### MO.4. What are the characteristics of plasma transfusion and its storage?

- Plasma of donors with identical ABO blood group to the recipient should be used as the first choice. If this is not possible, ABO non-identical but compatible plasma is acceptable if it has ‘low-titre’ anti-A or anti-B activity. (1B)
- Group O plasma should only be given to group O patients. (1B)
- FFP and cryoprecipitate of any RhD group may be transfused. If RhD positive plasma is given to an RhD negative individual, no anti-D prophylaxis is required. (1B)
- Once thawed, standard FFP or methylene blue treated FFP (MBFFP) may be stored at 2–4 °C in an approved temperature-controlled blood storage refrigerator before administration to the patient, as long as the infusion is completed within 24 h of thawing. (2A)
- Transfusion of FFP should be completed within 4 h of issue out of a controlled temperature environment. (2A)
- Pre-thawed FFP that is out of a controlled temperature environment 2–4 °C) can be accepted back into temperature- controlled storage if this occurs on one occasion only of less than 30 min). (2A)

#### Part IV: Acute transfusion reactions

The International Society of Blood Transfusion (ISBT) defines adverse transfusion reactions as undesirable responses or effects in patients temporarily associated with the administration of blood or blood components, and range in severity from minor reactions to reactions with a fatal outcome [38]. To minimize the risk of harm, early identification of reactions, rapid clinical assessment, and management are essential (Implementation tools Figure S4 and Figure S5).

##### TR.1. How to diagnose acute transfusion reaction (ATR)?

- All patients should be transfused in clinical areas where they can be directly observed, and where staff are trained in the administration of blood components and the management of transfused patients, including the emergency treatment of anaphylaxis. (1C)
- The recognition and immediate management of ATR should be incorporated into local transfusion policies and there should be mandatory transfusion training requirements for all clinical and laboratory staff involved in the transfusion process. (2C)
- If a patient develops new symptoms or signs during a transfusion, this should be stopped temporarily, but venous access maintained. Identification details should be checked between the patient, their identity band, and the compatibility label of the blood component. Perform visual inspection of the component and assess the patient with standard observations. (1C)
- Standard observations during blood components administration include the patient’s pulse rate, blood pressure, temperature, and respiratory rate should be monitored and abnormal clinical features, such as fever, rashes, or angioedema, frequently assessed. (GPP)

Symptoms and signs of acute transfusion reactions include:

- Fever and related inflammatory symptoms or signs, such as chills, rigors, myalgia, nausea, or vomiting.
- Cutaneous symptoms and signs including urticaria (hives), other skin rashes, and pruritus.
- Angioedema (localized oedema of the subcutaneous or submucosal tissues), which may be preceded by tingling.
- Respiratory symptoms and signs including dyspnea, stridor, wheeze, and hypoxia.
- Hypotension.
- Pain.
- Severe anxiety or ‘feeling of impending doom’.
- Bleeding diathesis with acute onset.
- If a patient develops sustained febrile symptoms or signs of moderate severity (temperature ≥ 39 °C OR a rise of ≥ 2 °C from baseline AND/OR systemic symptoms, such as chills, rigors, myalgia, nausea, or vomiting), bacterial contamination, or a hemolytic reaction should be considered. (1C)
- Patients should be asked to report symptoms that develop within 24 h of completion of the transfusion. (2C)
- If a patient being transfused for hemorrhage develops hypotension, careful clinical risk assessment is required. If the hypotension is caused by hemorrhage, continuation of the transfusion may be lifesaving. In contrast, if the blood component is considered the most likely cause of hypotension, the transfusion must be stopped or switched to an alternative component and appropriate management and investigation commenced. (1C)

##### TR.2. How to treat acute transfusion reactions?

- Initial treatment of ATR is not dependent on classification but should be directed by symptoms and signs. Treatment of severe reactions should not be delayed until the results of investigations are available. (1C)
- For patients with mild reactions, such as pyrexia (temperature of ≥ 38 °C AND rise of 1–2 °C from baseline), and/or pruritus or rash but WITHOUT other features, the transfusion may be continued with appropriate treatment and direct observation. (2B)
- Patients with mild isolated febrile reactions may be treated with oral paracetamol (500–1000 mg in adults). Patients with mild allergic reactions may be managed by slowing the transfusion and treatment with an antihistamine. (2C)
- Anaphylaxis should be treated with intramuscular adrenaline (epinephrine). Patients who are thrombocytopenic or who have deranged coagulation should also receive IM adrenaline if they have an anaphylactic reaction. (1A)

##### Shock/severe hypotension associated with wheeze or stridor

- For children over 12 years, administer IM adrenaline: 0.5 ml of 1:1000 adrenaline (500 lg) into the anterolateral aspect of the middle third of the thigh.
- For children between 6 and 12 years give 0.3 ml of 1:1000 IM adrenaline (300 lg).
- For children < 6 years give 0.15 ml of 1:1000 IM adrenaline (150 lg).
- Adrenaline is repeated, if necessary, at 5-min intervals according to blood pressure, pulse and respiratory function under the direction of appropriately trained clinicians.
- Supportive care of anaphylaxis includes:
- Rapid fluid challenge of 500–1000 ml crystalloid.
- Administration of 10 mg of chlorphenamine IM or by slow intravenous (IV) injection following initial resuscitation.
- Administration of 200 mg of hydrocortisone IM or by slow IV injection following initial resuscitation.
- If the patient has continuing symptoms of asthma or wheeze, inhaled or intravenous bronchodilator therapy should be considered.

(GPP)

##### Shock/severe hypotension without clinical signs of anaphylaxis or fluid overload

- Consider ABO incompatibility or bacterial contamination.
- Both require supportive care with fluid resuscitation, expert evaluation for inotropic, renal and/or respiratory support, and blood component therapy for disseminated intravascular coagulation with bleeding.
- If bacterial contamination is suspected, take blood cultures from the patient (peripheral vein and through central line, if present) and start broad-spectrum IV antibiotics (the local regimen for patients with neutropenic sepsis would be appropriate). Immediately notify the transfusion laboratory staff and hematologist to arrange culture of the implicated unit/units and contact the blood service so that any other components from the implicated donation can be recalled and quarantined.

(GPP)

##### Severe dyspnea without shock

- Consider transfusion-related acute lung injury (TRALI) or transfusion-associated circulatory overload (TACO). Ensure the airway is patent and high-flow oxygen therapy started while urgent expert medical assessment is obtained. Initial investigation should include chest X-ray and oxygen saturation.
- Primary treatment of TRALI is ventilatory support.

(GPP)

##### TR.3. What are the laboratory investigations done for acute transfusion reactions?

- In all moderate and severe transfusion reactions, standard investigations, including full blood count, renal and liver function tests, and assessment of the urine for hemoglobin should be performed. (2C)
- If febrile symptoms of moderate severity are sustained, implicated units should be returned to the laboratory for further investigation, the blood service contacted immediately so that associated components from the implicated donation can be withdrawn and the patient sampled for repeat compatibility and culture. (1C)
- Patients who have experienced moderate or severe allergic reactions should have IgA levels measured. Patients with IgA deficiency diagnosed after an ATR should be discussed with an allergist or immunologist regarding future management. (2C)

##### TR.4. What is the subsequent management of recurrent reactions?

- For patients with recurrent febrile reactions, it is recommended to give a trial of premedication with oral paracetamol 1 h before the reaction is anticipated (or non-steroidal anti-inflammatory drugs in patients with predominant chills or rigors—but an assessment of the risks of medication against the severity of reaction should be made in each case). Patients who continue to react should have a trial of washed blood components. (2C)
- For recurrent mild allergic reactions, there is no evidence to support routine prophylaxis with antihistamines or steroids. Alternative causes, such as allergy to drugs or latex gloves, should be excluded. (2C)
- For patients with recurrent moderate or severe allergic reactions, other than those in which the patient is IgA-deficient, options for further transfusion include:
- Use of directly monitored transfusion of standard components in a clinical area with resuscitation facilities. Consider antihistamine prophylaxis (although the evidence for efficacy is low, the risks are also low). This may be the only option when further transfusion is urgent and withholding blood is a greater risk. (2C)
- Transfusion of washed red cells or platelets. (2C)
- The use of pooled solvent detergent treated FFP when there are recurrent allergic reactions to FFP in patients undergoing plasma exchange. (2B)
- Patients with confirmed IgA deficiency and a history of reaction to blood should be transfused with components from IgA-deficient donors (first choice) or washed red cells (second choice) if time allows. (1C)
- Life-saving transfusion should not be denied or delayed if these are not immediately available but the facilities and skills to manage severe allergic reactions must be present. (1C)

##### TR.5. Do transfusion reactions need to be reported?

All transfusion reactions except mild febrile and/or allergic reactions must be reported to appropriate regulatory and hemovigilance organizations and should also be reviewed within the hospital. (1C).

## Summary

The goal of this work was to adapt global CPGs and their recommendations to the Egyptian healthcare setting for the comprehensive management of transfusion of blood components in different pediatric age groups across all local healthcare sectors.

The guideline covered all important aspects of the indications, dosing, and administration of packed red cells, platelets, and fresh frozen plasma. It also included transfusion in special situations, recommendations for safe transfusion practices, and recommendations for modification of cellular blood components. The final version of the adapted clinical practice guideline (CPG) has been made after a thorough review by an external review panel and was guided by their official recommendations and modifications.

A strength of this study is the use of the ‘Adapted ADAPTE’ method because it is structured and easy to follow, with a set of tools to support the process.

## Conclusion

This adapted guideline serves as a tool for safe transfusion practices in different pediatric age groups. The adaptation of CPG recommendations is a good and valid alternative to de novo developing a CPG for transfusion in pediatric age groups, especially given the lack of relevant, high-quality systematic reviews and randomized controlled trials in the Egyptian context.

### Supplementary Information

Below is the link to the electronic supplementary material.

Supplementary file1 (DOCX 28 KB)

Supplementary file2 (DOCX 1182 KB)

## Data Availability

Data will be available upon request.
