# Supplementary material for: Transfusion of blood components in pediatric age groups: an evidence-based clinical practice guideline adapted for the use in Egypt using ‘Adapted ADAPTE’
Source: Ann Hematol. 2024 Feb 22;103(4):1373–88. doi: 10.1007/s00277-024-05657-4 (PMC10940419; doi:10.1007/s00277-024-05657-4)
Supplement: Supplementary file 1 — Supplementary file1 (DOCX 28 KB) [file 277_2024_5657_MOESM1_ESM.docx]

## Table 1. The RIGHT-Ad@pt checklist

| 7 sections, 27 topics, and 34 items | | **Assessment** | **Page(s)*** | **Note(s)** | |
| --- | --- | --- | --- | --- | --- |
| **BASIC INFORMATION** | | | | |  |
| **Title/subtitle** | | | | |  |
| 1 | Identify the report as an adaptation of practice guideline(s), that is include "guideline adaptation", "adapting", "adapted guideline/recommendation(s)", or similar terminology in the title/subtitle. | Yes  No  Unclear |  |  | |
| 2 | Describe the topic/focus/scope of the adapted guideline. | Yes  No  Unclear |  |  | |
| **Cover/first page** | | | | |  |
| 3 | Report the respective dates of publication and the literature search of the adapted guideline. | Yes  No  Unclear |  |  | |
| 4 | Describe the developer and country/region of the adapted guideline. | Yes  No  Unclear |  |  | |
| **Executive summary/abstract** | | | | |  |
| 5 | Provide a summary of the recommendations contained in the adapted guideline. | Yes  No  Unclear |  |  | |
| **Abbreviations and acronyms** | | | | |  |
| 6 | Define key terms and provide a list of abbreviations and acronyms (if applicable). | Yes  No  Unclear |  |  | |
| **Contact information of the guideline adaptation group** | | | | |  |
| 7 | Report the contact information of the developer of the adapted guideline. | Yes  No  Unclear |  |  | |
| **SCOPE** | | | | |  |
| **Source guideline(s)** | | | | |  |
| 8 | Report the name and year of publication of the source guideline(s), provide the citation(s), and whether source authors were contacted. | Yes  No  Unclear |  |  | |
| **Brief description of the health problem(s)** | | | | |  |
| 9 | Provide the basic epidemiological information about the problem (including the associated burden), health systems relevant issues, and note any relevant differences compared to the source guideline(s). | Yes  No  Unclear |  |  | |
| **Aim(s) and specific objectives** | | | | |  |
| 10 | Describe the aim(s) of the adapted guideline and specific objectives, and note any relevant differences compared to the source guideline(s). | Yes  No  Unclear |  |  | |
| **Target population(s)** | | | | |  |
| 11 | Describe the target population(s) and subgroup(s) (if applicable) to which the recommendation(s) is addressed in the adapted guideline, and note any relevant differences compared to the source guideline(s). | Yes  No  Unclear |  |  | |
| **End-users and settings** | | | | |  |
| 12 | Describe the intended target users of the adapted guideline, and note any relevant differences compared to the source guideline(s). | Yes  No  Unclear |  |  | |
| 13 | Describe the setting(s) for which the adapted guideline is intended, and note any relevant differences compared to the source guideline(s). | Yes  No  Unclear |  |  | |
| **RIGOR OF DEVELOPMENT** | | | | |  |
| **Guideline adaptation group** | | | | |  |
| 14 | List all contributors to the guideline adaptation process and describe their selection process and responsibilities. | Yes  No  Unclear |  |  | |
| **Adaptation framework/methodology** | | | | |  |
| 15 | Report which framework or methodology was used in the guideline adaptation process. | Yes  No  Unclear |  |  | |
| **Source guideline(s)** | | | | |  |
| 16 | Describe how the specific source guideline(s) was(were) selected. | Yes  No  Unclear |  |  | |
| **Key questions** | | | | |  |
| 17 | State the key questions of the adapted guideline using a structured format, such as PICO (population, intervention, comparator, and outcome), or another format as appropriate. | Yes  No  Unclear |  |  | |
| 18 | Describe how the key questions were developed/modified, and/or prioritized. | Yes  No  Unclear |  |  | |
| **Source recommendation(s)** | | | | |  |
| 19 | Describe how the recommendation(s) from the source guideline(s) was(were) assessed with respect to the evidence considered for the different criteria, the judgements and considerations made by the original panel. | Yes  No  Unclear |  |  | |
| **Evidence synthesis** | | | | |  |
| 20 | Indicate whether the adapted recommendation(s) is/are based on existing evidence from the source guideline(s), and/or additional evidence. | Yes  No  Unclear |  |  | |
| 21 | If new research evidence was used, describe how it was identified and assessed. | Yes  No  Unclear | NA |  | |
| **Assessment of the certainty of the body of evidence and strength of recommendation** | | | | |  |
| 22 | Describe the approach used to assess the certainty/quality of the body/ies of evidence and the strength of recommendations in the adapted guideline and note any differences (if applicable) compared to the source guideline(s). | Yes  No  Unclear | NA |  | |
| **Decision-making processes** | | | | |  |
| 23 | Describe the processes used by the guideline adaptation group to make decisions, particularly the formulation of recommendations. | Yes  No  Unclear |  |  | |
| **RECOMMENDATIONS** | | | | |  |
| **Recommendations** | | | | |  |
| 24 | Report recommendations and indicate whether they were adapted, adopted, or *de novo*. | Yes  No  Unclear |  |  | |
| 25 | Indicate the direction and strength of the recommendations and the certainty/quality of the supporting evidence and note any differences compared to the source recommendations(s) (if applicable). | Yes  No  Unclear |  |  | |
| 26 | Present separate recommendations for important subgroups if the evidence suggests important differences in factors influencing recommendations and note any differences compared to the source recommendations(s) (If applicable). | Yes  No  Unclear |  |  | |
| **Rationale/explanation for recommendations** | | | | |  |
| 27 | Describe the criteria/factors that were considered to formulate the recommendations or note any relevant differences compared to the source guideline(s) (if applicable). | Yes  No  Unclear |  |  | |
| **EXTERNAL REVIEW AND QUALITY ASSURANCE** | | | | |  |
| **External review** | | | | |  |
| 28 | Indicate whether the adapted guideline underwent an independent external review. If yes, describe the process. | Yes  No  Unclear |  |  | |
| **Organizational approval** | | | | |  |
| 29 | Indicate whether the adapted guideline obtained organizational approval. If yes, describe the process. | Yes  No  Unclear |  |  | |
| **FUNDING, DECLARATION, AND MANAGEMENT OF INTEREST** | | | | |  |
| **Funding source(s) and funder role(s)** | | | | |  |
| 30 | Report all sources of funding for the adapted guideline and source guideline(s), and the role of the funders. | Yes  No  Unclear |  |  | |
| **Declaration and management of interests** | | | | |  |
| 31 | Report all conflicts of interest of the adapted and the source guideline(s) panels, and how they were evaluated and managed. | Yes  No  Unclear |  |  | |
| **OTHER INFORMATION** | | | | |  |
| **Implementation** | | | | |  |
| 32 | Describe the potential barriers and strategies for implementing the recommendations (if applicable). | Yes  No  Unclear |  |  | |
| **Update** | | | | |  |
| 33 | Briefly describe the strategy for updating the adapted guideline (if applicable). | Yes  No  Unclear |  |  | |
| **Limitations and suggestions for further research** | | | | |  |
| 34 | Describe the challenges of the adaptation process, the limitations of the evidence, and provide suggestions for future research. | Yes  No  Unclear | NA |  | |
