# Supplementary material for: Transfusion of blood components in pediatric age groups: an evidence-based clinical practice guideline adapted for the use in Egypt using ‘Adapted ADAPTE’
Source: Ann Hematol. 2024 Feb 22;103(4):1373–88. doi: 10.1007/s00277-024-05657-4 (PMC10940419; doi:10.1007/s00277-024-05657-4)
Supplement: Supplementary file 2 — Supplementary file2 (DOCX 1182 KB) [file 277_2024_5657_MOESM2_ESM.docx]

###### Supplementary material

###### Implementation Tools

The CPG adaptation group decided to adopt the implementation tools proposed including the following algorithms, pathways, tables, and parents’ educational health guide (in Arabic).


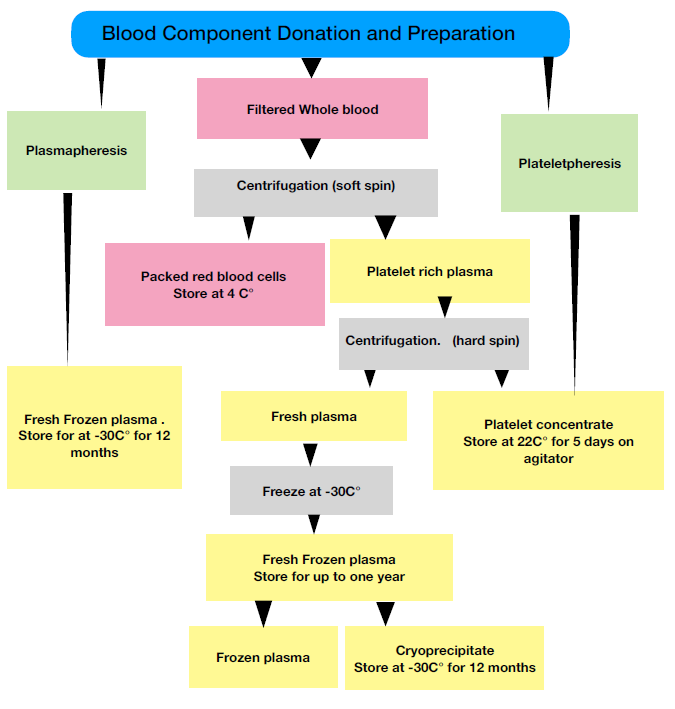


**Figure S1: The process by which whole blood is used to produce blood components and plasma derivatives.**

**Table S1: General transfusion practices**

| 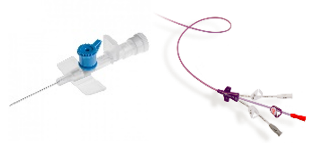 | **IV access**   - Blood components can be transfused through most peripheral or central venous catheters, although the flow rate is reduced by narrow lumen catheters and long peripherally inserted central catheters (PICC lines). |
| --- | --- |
| 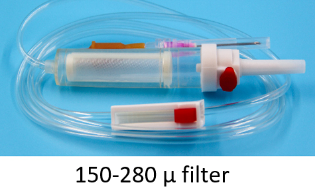 | **Administration sets and filters**   - They should be transfused through an administration set with a 170–200 μm integral mesh filter. - Pediatric administration sets with a smaller prime volume are available for small-volume transfusions. - Although special platelet administration sets are available, it is safe to use a standard blood administration set, but platelets should not be transfused through a set previously used for red cells as some platelet loss will occur. |
| 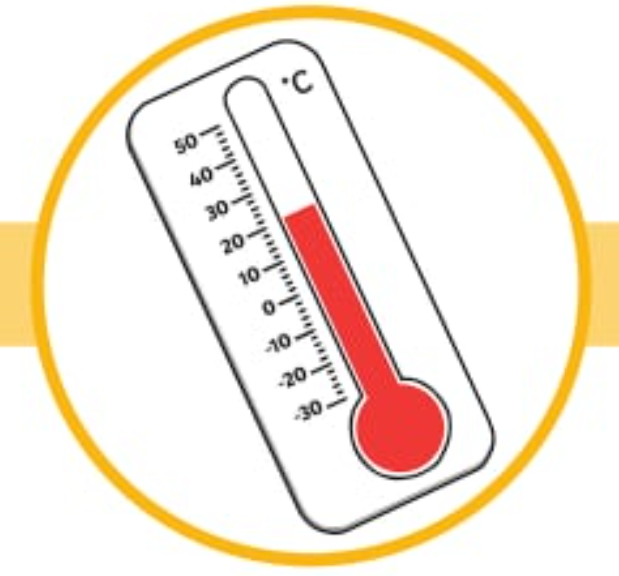 | **Blood warmers**   - Rapid infusion of red cells recently removed from the refrigerator may cause hypothermia. Concerns include impaired coagulation in surgical or trauma patients and cardiac arrhythmias if cold blood is transfused rapidly into a central catheter or in neonates and small infants having large-volume transfusions. The National Institute for Health and Care Excellence (NICE) in England recommends that, in all patients undergoing elective or emergency surgery, ‘intravenous fluids (500 mL or more) and blood products should be warmed to 37°C’. - Blood warmers may also be used in patients with clinically significant cold antibodies (discuss with a transfusion medicine specialist). |
| 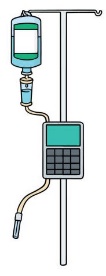 | **Infusion pumps**   - Data regarding the clinical effectiveness of intravenous infusion pumps for transfusion of blood products is lacking. - Red blood cell products can only be used with pumps certified for use with blood. |
| 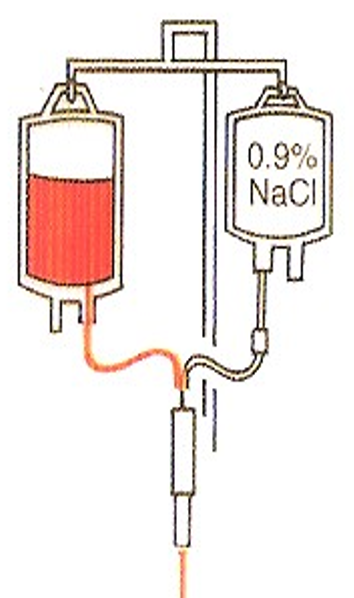 | **Compatible IV fluids**   - It is good practice to avoid the co-administration of any intravenous fluid through the same line used for blood components, unless a multi-lumen central venous catheter is used. Solutions containing calcium (e.g., Ringer’s lactate) or calcium-containing colloids antagonize citrate anticoagulant and may allow clots to form if mixed in the same infusion line. Hypotonic solutions, such as 5% dextrose in water, can cause hemolysis of red cells in laboratory experiments but the clinical significance of this is uncertain and no clinical adverse events have been reported. |
| 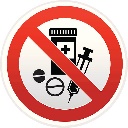 | **Concomitant administration of drugs**   - Drugs should never be added to a blood component bag. - Wherever possible, IV drugs should be administered between transfusions or administered through a second venous access device (or the separate lumen of a multi-lumen central venous catheter). If this is not possible, the transfusion should be temporarily stopped, and the line flushed with 0.9% saline before and after administration of the drug. |

|  | **Cut-off of platelet count** | | | | |  |
| --- | --- | --- | --- | --- | --- | --- |
|  | |  |  |  |  | |
| Less than 20 x10^3^/µl | |  | 20 x10^3^ to < 30 x10^3^/µl |  | 30 x10^3^ to 50 x10^3^/µl | |
|  | |  |  |  |  | |
| Mandatory transfusion | |  | Transfusion as clinical situation require (e.g., active bleeding, minor invasive procedure) |  | **Conditional transfusion in the following:**   - Intracranial hemorrhage - Coagulopathy - Low birth weight < 1000 g - Neonatal sepsis - FNAIT* - Major invasive procedure - Unstable blood pressure | |

*Washed irradiated platelets resuspended in ABO compatible plasma of maternal origin are used better. The transfused platelet should be lacking the causative antigen.

**Figure S2: Indication for platelet transfusion in neonates.**

**Table S2: Indications of Prophylactic Platelet Transfusion in Pediatrics**

| **Indication** | **Platelet threshold (x10^3^/µl)** |
| --- | --- |
| **Prior To Procedures or Surgery*** |  |
| - Major surgery or invasive procedure, no active bleeding | < 50 |
| - Venous central lines (both tunneled and un-tunneled) ** | <20 |
| - Lumbar puncture | < 40 |
| - Insertion/removal of epidural catheter | < 80 |
| - Major surgery | <50 |
| - Neurosurgery or ophthalmic surgery involving the posterior segment of the eye | <100 |
| - Percutaneous liver biopsy*** | <50 |
| **In critically ill child and reversible bone marrow failure** | <10 |
| **In critically ill child and reversible bone marrow failure with additional risk factors for bleeding****** | <20 |
| **In Stable, non-bleeding child** | <10 |

* Whenever possible use a procedure/equipment associated with the lowest bleeding risk. Apply local measures, such as compression, to reduce the risk of bleeding post-procedure.

** Inserted by experienced staff using ultrasound guidance techniques.

***Consider trans-jugular biopsy if the platelet count is below this level.

****e.g., body temperature > 38°C or undergoing invasive procedure.


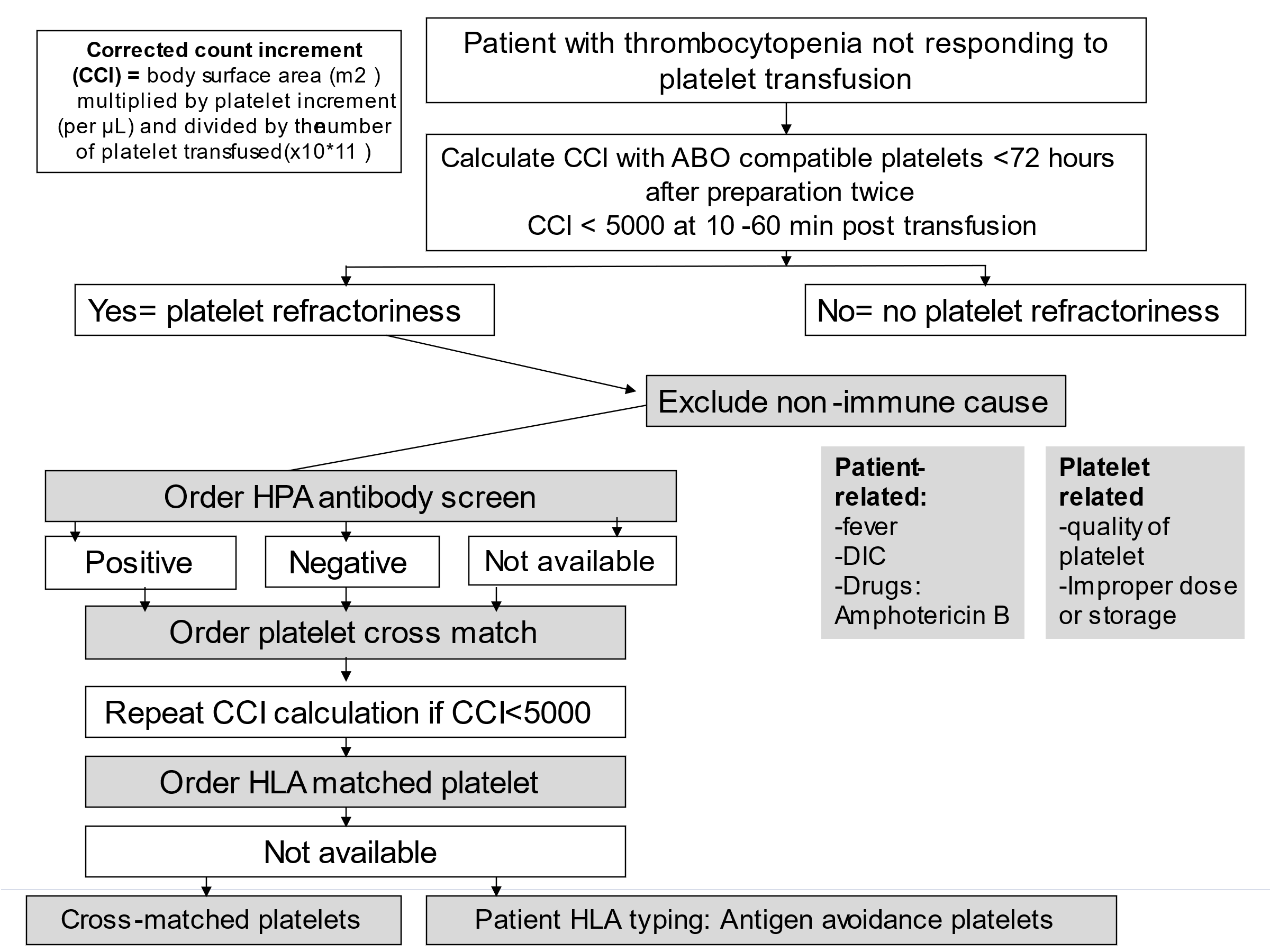


**Figure S3: Approach to Platelet Refractoriness**

HPA: human platelet antigen, HLA: human leukocyte antigen

**Table S3: Indications of fresh frozen plasma transfusion**

| **Indications** | **Associated conditions** |
| --- | --- |
| Isolated Coagulation factors deficiency | When specific therapy (recombinant or viral inactivated product) is not available |
| Multiple coagulation factor deficiencies | As DIC, vitamin k deficiency and liver failure If there is active bleeding in addition to treatment of the cause |
| Anticoagulant | - Reversal of warfarin (Coumadin)- with active bleeding or require emergency surgery. - Other anticoagulants: before surgery to prevent active bleeding or to treat active bleeding |
| Microvascular bleeding | When PT and PTT> 1.5 times normal |
| Thrombotic thrombo-cytopenic purpura | Therapeutic plasma exchange |
| Protein C & protein S deficiency | Associated with hypercoagulable state and no available concentrates |

**Table S4: Indication and dose of cryoprecipitate in various coagulation disorders**

| **Indications** | **Dose of cryoprecipitate** |
| --- | --- |
| **Fibrinogen Replacement** | - Cryoprecipitate can be used for fibrinogen replacement in fibrinogen disorders (congenital afibrinogenemia or dysfibrinogenemia) - Dose: 1 unit of cryoprecipitate per 5 kg patient weight will increase fibrinogen by about 100 mg/dL - Number of bags = 0.2 x weight (kg) to provide about 100 mg/dL fibrinogen. |
| **Factor XIII Replacement** | - 1 unit of cryoprecipitate per 5kg patient weight will provide 10 U/kg of factor XIII. - Number of bags =0.2 x weight (kg). - Factor XIII has a long half-life and can usually be dosed every 3-6 weeks. |
| **Factor VIII Replacement** | - Dosing depends on patient factor VIII (8) level and requires routine monitoring of factor VIII (8) to determine appropriate dose. - Dosing should be repeated every 8-12 hours but will vary with each patient. - Post-surgery or major trauma replacement may be required for up to 10 days to maintain hemostasis. - Dosing also depends on Plasma Volume (PV) which is a fraction of Total Blood Volume (TBV). - Number of bags = [Desired activity (%) – Current activity (%)] x PV / 80   *N.B:* *PV (mL) = TBV x (1-Hct), TBV (mL) = 70 mL/kg x weight (kg)* |
| **Von Willebrand Factor Replacement** | - Dosing of 1 unit per 10 kg patient weight will usually be enough to control bleeding. - Number of bags = 0.1 x weight (kg) - Repeat dosing may be required every 8-12 hours for up to 3 days followed by once daily dosing. - Follow clinically to adjust dosing and with appropriate lab studies available at your institution. |


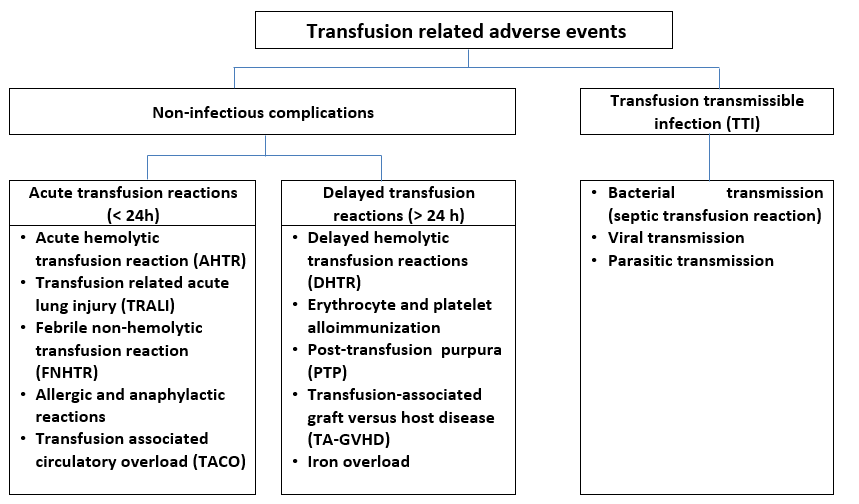


**Figure S4: Different types of transfusion related adverse events.**

**
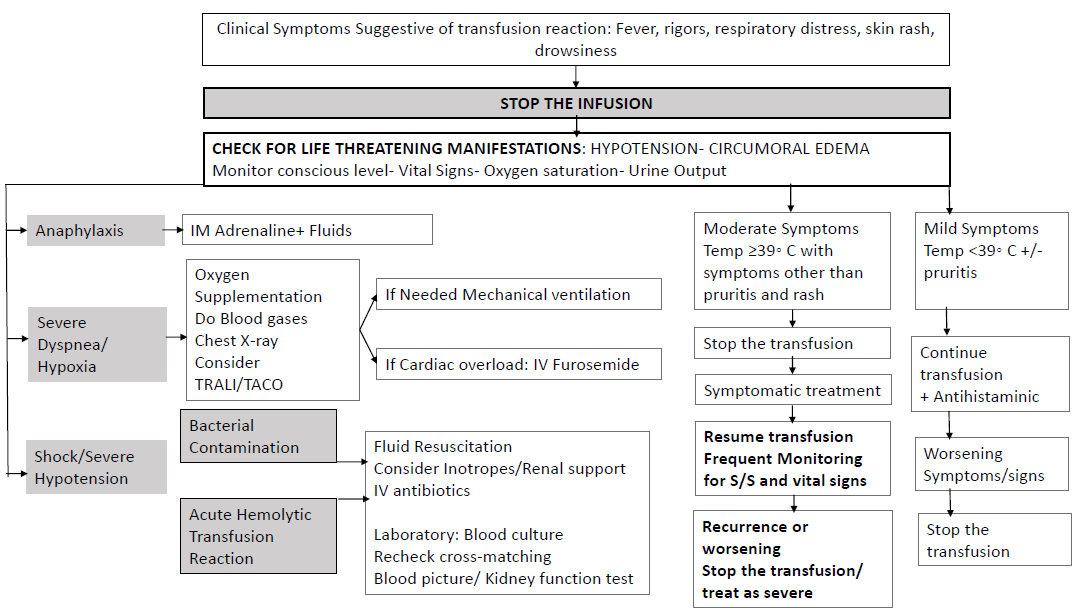
**

**Figure S5: Management of acute transfusion reactions.**
